# CAvant® WO-60 as an Effective Immunological Adjuvant for Avian Influenza and Newcastle Disease Vaccine

**DOI:** 10.3389/fvets.2021.730700

**Published:** 2021-12-03

**Authors:** Eun-Seo Lee, Young-Jung Shim, W. A. Gayan Chathuranga, Young-Hoon Ahn, In-Joong Yoon, Sung-Sik Yoo, Jong-Soo Lee

**Affiliations:** ^1^College of Veterinary Medicine, Chungnam National University, Daejeon, South Korea; ^2^Choong Ang Vaccine Laboratory Co., Ltd., Daejeon, South Korea

**Keywords:** avian influenza virus, Newcastle disease virus, vaccine, adjuvant, water-in-oil emulsion, CAvant® WO-60

## Abstract

Despite the immunogenicity of vaccines currently used in poultry, several pathogens, including avian influenza virus (AIV) and Newcastle disease virus (NDV), cause enormous economic losses to the global poultry industry. The efficacy of vaccines can be improved by the introduction of effective adjuvants. This study evaluated a novel water-in-oil emulsion adjuvant, CAvant® WO-60, which effectively enhanced both the immunogenicity of conserved influenza antigen sM2HA2 and inactivated whole H9N2 antigen (iH9N2). CAvant® WO-60 induced both humoral and cell-mediated immunity in mice and provided 100% protection from challenge with 10 LD50 of A/Aquatic bird/Korea/W81/2005 (H5N2) and A/Chicken/Korea/116/2004 (H9N2) AIV. Importantly, immunization of chickens with iH9N2 plus inactivated NDV LaSota (iNDV) bivalent inactivated vaccine emulsified in CAvant® WO-60 induced seroprotective levels of antigen-specific antibody responses. Taken together, these results suggested that CAvant® WO-60 is a promising adjuvant for poultry vaccines.

## Introduction

Avian influenza (AI) and Newcastle disease (ND) are two of the diseases that affect poultry, causing enormous economic losses to the poultry industry worldwide (1). AI results from infection with avian influenza viruses (AIVs), which belong to the genus Influenza virus A and the family Orthomyxoviridae (2). Although infection of poultry with AIVs can be asymptomatic, it can also induce various symptoms of disease, including respiratory illnesses, reduced egg production, and severe systemic diseases with near 100% mortality rates (3, 4). AIVs can be further divided into two categories, low pathogenic avian influenza (LPAI) and high pathogenic avian influenza (HPAI), based on their genetic features and pathogenicity (4, 5). All naturally occurring HPAI strains isolated to date have been either of the H5 or H7 subtype (2).

ND results from infection with avian paramyxovirus serotype 1 (APMV-1), also called Newcastle disease virus (NDV). This virus belongs to the genus Avulavirus and the family Paramyxoviridae. NDV infection can be asymptomatic in poultry, but it can also induce disease symptoms, including depression, prostration, diarrhea, and nervous signs, with nearly 100% mortality rates (6). Based on the clinical signs in infected chickens, NDV has been classified into four categories, the velogenic, mesogenic, lentogenic, and asymptomatic pathotypes (7). Co-infection of poultry with NDV and LPAIV–H9N2 may lead to severe clinical complications, with a higher mortality rate when compared with infection with a single virus (1, 8, 9).

Inactivated AIV and NDV antigens have been prophylactically included in water-in-oil (W/O) emulsion vaccines to control widespread outbreaks of AI and ND in enzootic countries (10). To be effective, vaccines require an appropriate antigen that matches the challenging virus. This remains a challenge in formulating AIV vaccines because AIVs often undergo mutations that alter their antigenicity (11). Furthermore, HPAIV cannot be used as a seed virus for the production of vaccines. The inability to use HPAIV as a seed virus and the difficulty predicting the antigenic shift of AIV indicate the need for new AIV vaccines based on epitopes common to various AIV subtypes and that can provide universal protection. Most universal vaccines currently under development are based on conserved epitopes in in matrix protein 2 (sM2), the stalk domain of HA (HA2), and other AIV structural proteins (12–14). A recombinant protein composed of both sM2 and HA2 (sM2HA2) has been shown to produce cross-reactive responses (15). However, the major limiting factor in the further development of vaccine remains the poor immunogenicity of antigens when administered alone (14, 16).

Attempts have been made to develop methodologies that will improve the production of cross-reactive antibodies and T-cell immune responses upon vaccination. These include the incorporation of vaccine adjuvants, consisting of chemical substances, microbial components, and/or proteins that enhance immune responses to vaccines. Adjuvants not only improve vaccine immunogenicity, but also can reduce the amount of antigen that must be administered, reduce the number of immunizations, and broaden immune responses to antigenically shifted variants. Although numerous commercial and experimental adjuvants have been tested in the last few decades, these adjuvants have limitations, including lack of efficacy, a tendency to induce systemic toxicity, manufacturing difficulties, poor stability, and high cost (17–19). For example, although aluminum-based mineral salts, the most widely used adjuvants in human and avian influenza vaccines, significantly enhance humoral responses to these viruses, their ability to enhance cellular immune responses is poor (20). Adjuvants are therefore needed that elicit both appropriate humoral immune responses and effective cellular immune responses to vaccines.

The present study evaluated a new W/O emulsion adjuvant, called CAvant® WO-60 (CAVAC, Korea), to determine whether it improves the immunogenicity of influenza antigens. The conserved recombinant sM2HA2 protein and the inactivated H9N2 (iH9N2) virus were emulsified in CAvant® WO-60 or the reference adjuvant ISA 70 VG (SEPPIC, France), followed by the immunization of mice with these vaccine formulations. CAvant® WO-60 was found to effectively enhance both the humoral and cellular immune responses of mice to these AIV vaccines. Moreover, immunization of mice with these vaccines in CAvant® WO-60 protected the mice from lethal AIV challenge. Furthermore, CAvant® WO-60 adjuvant contributed to the induction of seroprotective levels of antigen-specific hemagglutination inhibiting (HI) antibody responses to bivalent inactivated AIV-NDV vaccine in chicken. These findings suggest that CAvant® WO-60 may be a successful adjuvant for vaccines in poultry.

## Materials and Methods

### Preparation of Vaccines

The sM2HA2 protein comprising conserved matrix protein 2 (sM2) and stalk domain of hemagglutinin (HA2) was constructed and purified as previously described (15). Briefly, Sequence verified synthesized sM2HA2 (662 bp) gene fragment was inserted into the multiple cloning site of the pRSETA vector (Invitrogen, USA) using *Bam*HI and *Eco*RI restriction enzyme sites. The recombinant 6xHis-M2e fusion protein was expressed in *E. coli* BL2-CodonPlus (DE3)-RIPL chemically competent cells purified by Fast protein liquid chromatography (FPLC) using immobilized metal affinity chromatography (IMAC) column (Bio-Rad, USA). The purified proteins were dialyzed using a permeable cellulose membrane (molecular mass cutoff, 12–14 kDa; Spectrum Laboratories, Auckland, New Zealand) in PBS at 4°C. The protein concentration was measured using Bradford assays (Bio-Rad, Hercules, CA). iH9N2 and inactivated NDV (iNDV) antigen formulation were performed according to the Office International des Épizooties (OIE) manual of diagnostic tests and vaccines for terrestrial animals (21). Briefly, the virus A/Chicken/Korea/116/2004(H9N2) (A/Chicken/Korea/01310/2001) and NDV (LaSota) were propagated in 10-day-old embryonated SPF chicken eggs. To determine the 50% egg infectious dose (EID50) of the propagated virus, eggs were inoculated with serially diluted virus and EID50 was calculated using the Reed and Muench method (22). The virus was inactivated using 0.2% formalin as described previously (23). The inactivated viruses were then inoculated into the 10 day old embryonated SPF chicken eggs to confirm the virus inactivation. Mineral oil-based adjuvant Montanide ISA 70 VG was purchased from SEPPIC (Paris, France); Mineral oil-based CAvant® WO-60 adjuvant was newly developed by Choong Ang Vaccine Laboratories Company (Daejeon, Korea) that forms low viscous (4.5 cP at 25°C) W/O microemulsions with small droplet size (<2 microns). For mice immunization, antigen loads of sM2HA2 15 μg/head and iH9N2 10^7^ EID_50_/dose were emulsified either with ISA 70 VG or newly formulated adjuvant CAvant® WO-60 at a 3:7 ratio (v/v) using a high shear mixer (Primix, Japan). For chicken immunization, inactivated NDV (LaSota) and LPAI (A/chicken/Korea/01310/2001) containing bivalent antigen and also 10^8^ EID_50_/dose from each antigen were emulsified either with ISA 70 VG or with CAvant® WO-60 at a 3:7 ratio (v/v).

### Mice, Immunization, Virus Challenge, and Sample Collection

The specific pathogen-free (SPF) female BALB/c mice (6 weeks) were purchased from Samtako (Seoul, Korea) and housed in a temperature- and light-controlled milieu and had free access to food and water. In terms of mouse experiments, they were divided into two experiment regimes (one for the conserved influenza sM2HA2 antigen immunization and the other for iH9N2 antigen immunization) with four groups each. Each regime had 16 mice per group [5 for humoral and cellular immune responses evaluation, 6 for lung virus titration at 3 and 5 days post-infection (DPI), and 5 for body weight changes and survival rate screening after lethal challenge]. In specificity, groups of mice were intramuscularly (i.m.) immunized with PBS, sM2HA2, and sM2HA2 plus ISA 70 VG, and sM2HA2 plus CAvant® WO-60 emulsion. In each group, the dose of sM2HA2 antigen vaccinated into mice was kept constant at 15 μg/head in the total volume of 100 μl at the caudal thigh muscle of both hind limbs with 50 μl per hind limb. Mice were immunized twice every other week. Similarly, groups of other sets of mice were i.m. immunized with PBS, iH9N2, iH9N2 plus ISA 70 VG, and iH9N2 plus CAvant® WO-60 following the same immunizing schedule. The antigen dose of inactivated iH9N2 was kept constant at 10^7^ EID_50_/dose in the total volume of 100 μl at the caudal thigh muscle of both hind limbs with 50 μl per hind limb (Table 1).

**Table 1:** Groups for the mouse experiments.

**Regime**	**Vaccine group**		**Route**	**No. of mice tested in each group**		
	**Antigen**	**Adjuvant**		**ELISPOT and ELISA**	**Lung virus titer (3 and 5 DPI)**	**Survival and body weight**
01	–	PBS	i.m.	5	3 for each time	5
	sM2HA2	–		5	3 for each time	5
	sM2HA2	ISA 70 VG		5	3 for each time	5
	sM2HA2	CAvant® WO-60		5	3 for each time	5
02	–	PBS	i.m.	5	3 for each time	5
	iH9N2	–		5	3 for each time	5
	iH9N2	ISA 70 VG		5	3 for each time	5
	iH9N2	CAvant® WO-60		5	3 for each time	5

*PBS, phosphate buffered saline. i.m., intramuscular. DPI, day post-infection*.

**Figure 1:**
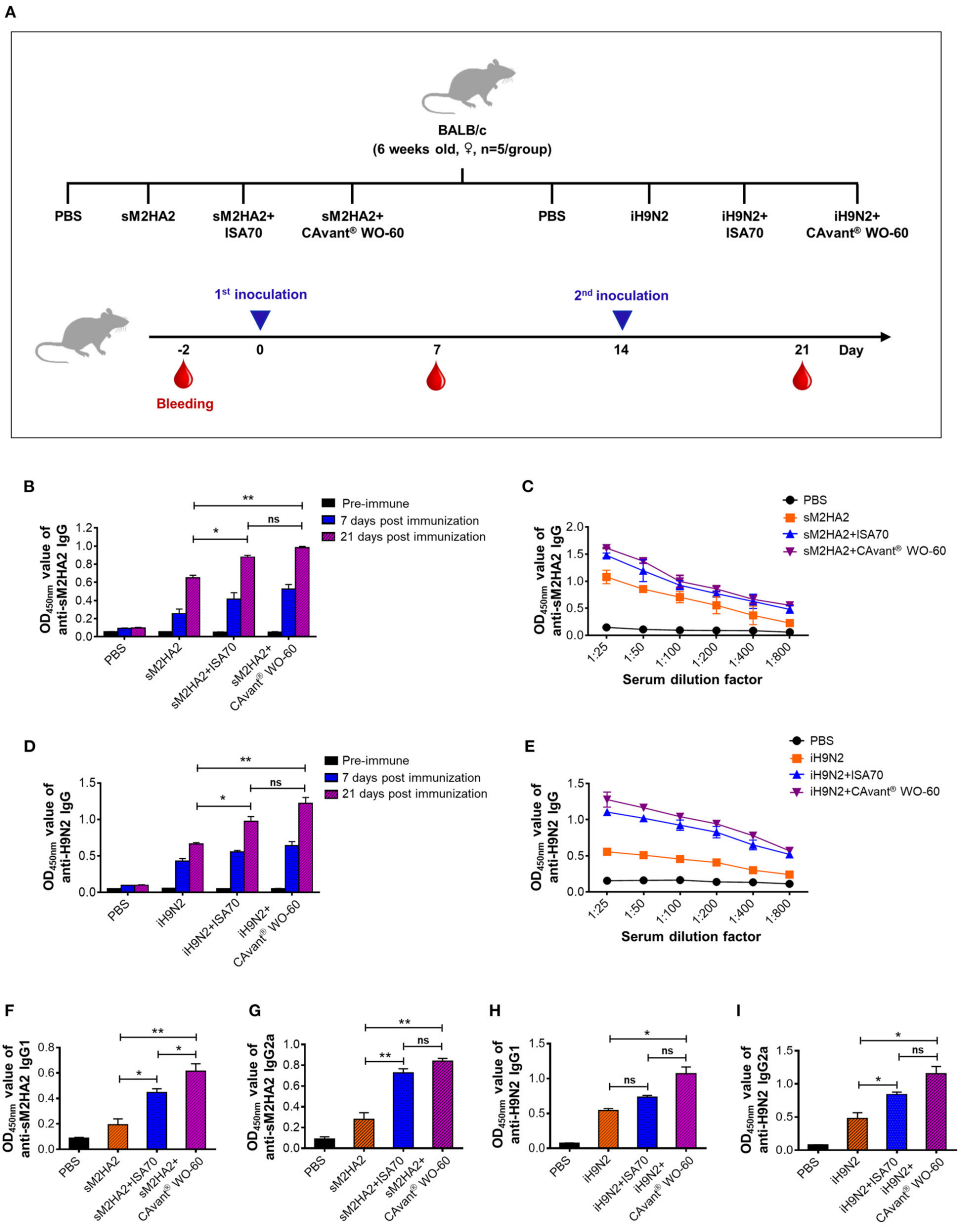
Evaluation of the antigen-specific humoral immune response in mice. **(A)** Schematic depiction of mice experiment strategy. Mice were intramuscularly administered twice every other week. Serum samples were collected on day−2 (pre-serum), day 7, and day 21 after first immunization. The antibody response levels were detected by indirect ELISA. **(B)** Kinetics of appearance IgG at 1:100 serum dilution ratio and **(C)** comparative serum IgG antibody titers at 21 days post-immunization upon immunization with PBS, sM2HA2, sM2HA2+ISA 70 VG, and sM2HA2+CAvant® WO-60. **(D)** Kinetics of appearance IgG at 1:100 serum dilution ratio and **(E)** comparative serum IgG antibody titers at 21 days post-immunization upon immunization with PBS, iH9N2, iH9N2+ISA 70 VG, and iH9N2+CAvant® WO-60. **(F)** Systemic IgG1, **(G)** Systemic IgG2a antibody responses specific to sM2HA2 in the sera at day 21 after immunization with PBS, sM2HA2, sM2HA2+ISA 70 VG, and sM2HA2+ CAvant® WO-60. **(H)** Systemic IgG1, **(I)** Systemic IgG2a antibody responses specific to iH9N2 in the sera at day 21 after immunization with PBS, iH9N2, iH9N2+ISA 70 VG, and iH9N2+CAvant® WO-60. The bar shows the mean ± SD of *n* = 5 samples. Data from one representative experiment of two independent experiments are shown. Comparison of groups was analyzed by ANOVA and Tukey's multiple comparisons test. *p < 0.05, **p < 0.01 between CAvant® WO-60 adjuvanted, ISA 70 VG adjuvanted, and antigen-only group.

The mouse-adapted low-pathogenic AI, namely, A/Aquatic bird/Korea/W81/2005 (H5N2) and A/Chicken/Korea/116/2004(H9N2), were used in the challenge experiments, and these were generously supplied by Dr. Young-Ki Choi (College of Medicine and Medical Research Institute, Chungbuk National University, Cheongju, Republic of Korea). The mice were anesthetized by ether inhalation following intranasal infection with 10LD_50_ of H5N2 or H9N2 influenza A subtypes on day 28. The survival rate was determined by death or a cutoff of 25% lost body weight, at which point the animals were humanely euthanized. All efforts were made to minimize suffering, and all of the surviving mice were humanely euthanized using CO_2_ inhalation for 5 min after final monitoring. In all immunization groups, mouse sera were procured for antibody determination at the respective time points depicted in Figure 1A and were stored at−20°C until proceeding. Five mice from each group were sacrificed on day 24 after prime vaccination to obtain splenocytes for the analysis of antigen-specific T-cell responses.

### Chicken, Immunization, and Sample Collection

The total number of 95, 6 week old (Strain: Leghorn) SPF chickens, purchased from Namdeok SPF (Korea), were divided into three experiment regimes: one regime for *in vivo* safety profile of the CAvant® WO-60 that contained two groups and other two regime for single immunization and priming-boosting immunization, which contained three groups per regime. In an *in vivo* safety profile regime both test group and control group consisted of 5 birds, chickens in the test group were vaccinated intramuscularly with 500 μl of inoculum contained 10^8^ EID_50_/dose from Newcastle disease virus (LaSota) and 10^8^ EID_50_/dose from LPAIV (A/chicken/Korea/01310/2001) antigen emulsified with CAvant® WO-60, chickens in the control group were maintain without vaccination (Table 2). After vaccination, the chickens were observed for general clinical signs, loss of appetite, and respiratory and gastrointestinal abnormalities for 28 days. The bodyweight of the chickens was measured at 0, 1, 2, 3, and 4 weeks after vaccination. In a single immunization regime, each group consisted of 20 birds; the first and second groups were immunized with 500 μl of inoculum containing 10^8^ EID_50_/dose from NDV (LaSota) and 10^8^ EID_50_/dose from LPAIV (A/chicken/Korea/01310/2001) antigen emulsified either with ISA 70 VG or with CAvant® WO-60 *via* intramuscular injection into the breast muscle. The birds of the third group were inoculated with 500 μl of sterile PBS. Blood samples were collected 21 days after immunization from the brachial vein as depicted in **Figure 4A**. In a priming-boosting immunization regime, the first and second groups consisting of 10 birds and the control group consisting of 5 birds were immunized twice at 2 week intervals with the same inoculum composition that was used in a single immunization experiment. Peripheral blood was collected at different time points up to 34 weeks as depicted in **Figure 4B**. Blood was incubated at room temperature for 30 min, and serum was separated from the whole blood by centrifugation at 12,000 rpm for 5 min.

**Table 2:** Groups for the chicken experiments.

**Regime**	**Vaccine group**		**Route**	**No. of immunization**	**No. of chicken tested in each group**
	**Antigen**	**Adjuvant**			
01	–	–	i.m.	–	5
	iH9N2+iNDV	CAvant® WO-60		2	5
02	–	PBS	i.m.	1	20
	iH9N2+iNDV	ISA 70 VG		1	20
	iH9N2+iNDV	CAvant® WO-60		1	20
03	–	PBS	i.m.	2	5
	iH9N2+iNDV	ISA 70 VG		2	10
	iH9N2+iNDV	CAvant® WO-60		2	10

*iH9N2, Inactivated A/chicken/Korea/01310/2001 strain; iNDV, Inactivated Newcastle disease virus LaSota strain; PBS, phosphate buffered saline; i.m., intramuscular*.

### Enzyme-Linked Immunosorbent Assay (ELISA)

Ninety-six-well immunosorbent plates (Nunc, USA) were coated overnight at 4°C with 500 ng/well of sM2HA2 recombinant protein or with 1 μg/well of iH9N2 for serum IgG, IgG1, and IgG2a ELISA. After blocking for 2 h with 5% skim milk at room temperature (RT), serial twofold dilution of serum samples (1:25 to 1:800) was added into the wells, and the plates were incubated for 2 h at 37°C, treated with HRP-conjugated goat anti-mouse immunoglobulins (IgG, IgG1, and IgG2a, 1:3,000, Sigma, Korea) as the secondary antibodies, and incubated at 37°C for 2 h. The plates were then reacted with tetramethylbenzidine and H_2_O_2_-mixed substrate (BD Bioscience, USA) solutions for 10 min in the dark. Finally, the reactions were stopped by the addition of 2N-H_2_SO_4_, and the optical density (OD) values were measured at 450 nm using a scanning multi-well spectrophotometer (ELISA reader, Molecular Devices).

### Splenocyte Isolation, Stimulation, and Elispot

For the analysis of antigen-specific T-cell responses, briefly, BD ELISPOT 96-well plates were coated with anti-mouse IFN-γ or IL-4 capture antibodies in 100 μl of PBS/well and incubated at 4°C overnight. The plates were blocked with complete RPMI 1640 medium containing 10% fetal bovine serum (Gibco, USA) and incubated in RT for 2 h. Freshly isolated splenocytes were added at 1 × 10^6^ cells/well in media containing the 1 μg/well of sM2HA2 protein or M2 or HA2 peptide (Table 3) or 1 μg/well of inactivated H9N2 or only medium (negative control), or 0.5 μg/well phytohemagglutinin (positive control, Invitrogen, Carlsbad, CA, USA). Plates were incubated for 24 h for IFN-γ and 48 h for IL-4 at 37°C in 5% CO_2_. After discarding the cells, the plates were treated sequentially with biotinylated anti-mouse IFN-γ and IL-4 antibodies, streptavidin-HRP, and substrate solution. Finally, the plates were washed with deionized water and dried for at least 2 h in the dark. Spots were counted automatically using an Immuno Scan Entry analyzer (Cellular Technology Ltd., Shaker Heights, OH, USA).

**Table 3:** Peptides used for ELISPOT.

**Protein^a^**	**aa position**	**aa sequence**
HA2	19–48	GYAADLKSTQNAIDEITNKVNSVIEKMNTQ
M2	2–16	SLLTEVETPTRNEWE

a *Proteins of the A/EM/Korea/W149/06 (H5N1) virus. aa, Amino acid*.

### Lung Virus Titration

Fifty percent tissue culture infectious dose (TCID_50_) assays were performed to determine the virus titers in the lungs as previously described (21). Briefly, the lung tissues were homogenized in PBS containing an antibiotic and antimycotic solution (Gibco, USA) and centrifuged at 12,000 × *g* to remove cellular debris. Ten-fold serial dilutions of samples were added to the confluent MDCK cells at 37°C in a humid atmosphere for 1 h. An overlay medium containing L-1-tosylamide-2-phenylethyl chloromethyl ketone (TPCK) trypsin (Thermo Fisher Scientific, USA) was replaced without supplemented serum, and the infected cells were incubated for 48 h. After a cytopathic effect (CPE) was observed, a hemagglutination assay (HA) was performed, and the virus titers were calculated by the Reed and Muench method (23) and expressed as log_10_ TCID_50_/lung tissue.

### Hemagglutination Inhibition (HI) Test

Sera from vaccinated chicken were heat-inactivated at 56°C for 30 min. Fifty microliters of sera were two-fold serially diluted in duplicate in round-bottom 96-well plates and mixed with an equal volume of 4 HA units of either NDV (LaSota) at RT for 30 min. AIV(A/chicken/Korea/01310/2001) virus was performed in V-bottom 96-well plates. Fifty microliters of 1% chicken red blood cells was added to each well and incubated for 20 min at room temperature before plate reading.

### Statistical Analysis

Statistical analysis was performed using GraphPad Prism version 6 software (GraphPad Software). All quantitative data were expressed as standard errors of the mean (SEM) or geometric mean (GM). Statistical significance was assessed using ANOVA followed by Tukey's multiple comparisons test. Comparison of survival was performed by a log-rank test. *p*-values of < 0.05 were considered statistically significant.

## Results

### CAvant® WO-60 Adjuvant Improved Antigen-Specific Humoral Immune Responses to AIV Antigens in Mice

Groups of mice were immunized intramuscularly (i.m.) on days 0 and 14 with sM2HA2 protein (15 μg/dose) or iH9N2 (107 EID50/dose) emulsified 3:7 (v/v) in CAvant® WO-60 or ISA 70 VG adjuvant (v/v). Other groups of mice were injected i.m. with the protein or inactivated virus alone or with PBS. Serum samples collected immediately before immunization and 7 and 21 days after the first immunization were subjected to indirect ELISA using purified sM2HA2 and iH9N2 as the coating antigens (Figure 1A).

Seven days after the first immunization, the mice had relatively low sM2HA2-specific IgG levels, regardless of whether the antigens were delivered with or without an adjuvant. By contrast, 7 days after the second immunization (day 21), all mice that were injected with sM2HA2 exhibited high antigen-specific antibody levels. Moreover, anti-sM2HA2 IgG antibody titers were significantly higher in the sera of mice immunized with sM2HA2 emulsified in CAvant® WO-60 than in the sera of mice immunized with sM2HA2 alone (Figures 1B,C). Similar results were observed in mice immunized with iH9N2, in that second immunization boosted IgG responses to H9N2, with the highest H9N2-specific IgG titers observed in the sera of mice immunized with iH9N2 emulsified in CAvant® WO-60 (Figures 1D,E).

IgG isotype analysis by indirect ELISA using purified sM2HA2 protein and iH9N2 showed that antigen-specific IgG1 and IgG2a titers were balanced in the mice immunized with these antigens emulsified in CAvant® WO-60. Interestingly, antigen-specific IgG1 and IgG2a titers were higher in mice immunized with viral antigens emulsified in CAvant® WO-60 than in mice injected with antigens alone or emulsified in ISA 70 VG (Figures 1F–I). Thus, the results suggest that the newly introduced CAvant® WO-60 adjuvant enhanced humoral immune responses to AIV antigens.

### CAvant® WO-60 Adjuvant Improved Antigen-Specific T-Cell Responses to AIV Antigens in Mice

**Figure 2:**
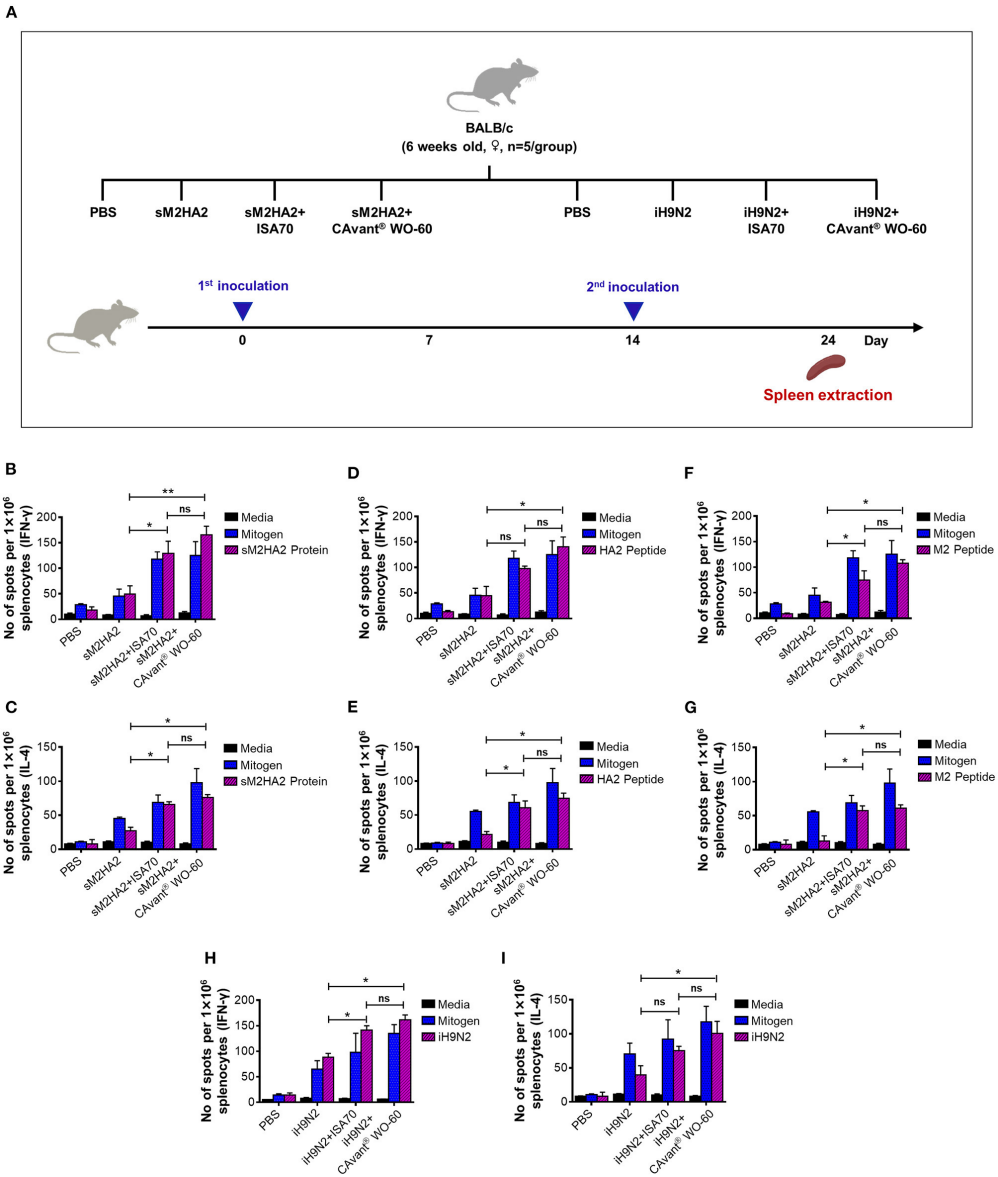
Evaluation of the cell-mediated immune responses in mice. **(A)** Schematic depiction of mice experiment strategy. Splenocytes were harvested 10 days after the last immunization. Cells were re-stimulated *in vitro* with the **(B,C)** sM2HA2 protein, **(D,E)** HA2 peptide, **(F,G)** M2 peptide, and **(H,I)** iH9N2 and IFN-γ and IL-4 spot forming cell were determined by ELISPOT assay. The bar shows the mean ± SD of *n* = 5 samples. Data from one representative experiment of two independent experiments are shown. Comparison of groups was analyzed by ANOVA and Tukey's multiple comparisons test. *p < 0.05, **p < 0.01 between CAvant® WO-60 adjuvanted, ISA 70 VG adjuvanted, and antigen-only group.

The above-described groups of mice were sacrificed 24 days after the first immunization and their splenocytes were subjected to ELISPOT assays to quantify the numbers of antigen-specific cells that secreted interferon (IFN)-γ and interleukin (IL)-4 (Figure 2A). IFN-γ is a representative Th1 cytokine that is also expressed by cytotoxic T lymphocytes, whereas IL-4 is a Th2 cytokine. Thus, the splenocytes from mice immunized with sM2HA2 were stimulated with sM2HA2 or M2 or HA2 peptides. The percentages of IFN-γ and IL-4-secreting splenocytes were higher in mice immunized with sM2HA2 emulsified in CAvant® WO-60 than in mice immunized with sM2HA2 alone or with sM2HA2 emulsified in ISA 70 VG (Figures 2B–G). Similar results were observed when the splenocytes from mice immunized with iH9N2 were stimulated with whole H9N2 antigen. The percentages of IFN-γ and IL-4-secreting splenocytes were again higher in mice immunized with iH9N2 emulsified in CAvant® WO-60 than in mice immunized with the inactivated virus alone or with iH9N2 emulsified in ISA 70 VG (Figures 2H,I). These results provide evidence that immunization with sM2HA2 emulsified in CAvant® WO-60 enhanced antigen-specific T-cell immune responses to a similar or even higher degree than immunization with sM2HA2 emulsified in the control adjuvant ISA 70 VG.

### CAvant® WO-60 Adjuvant Enhanced Protection Against AIV Infection in Mice

**Figure 3:**
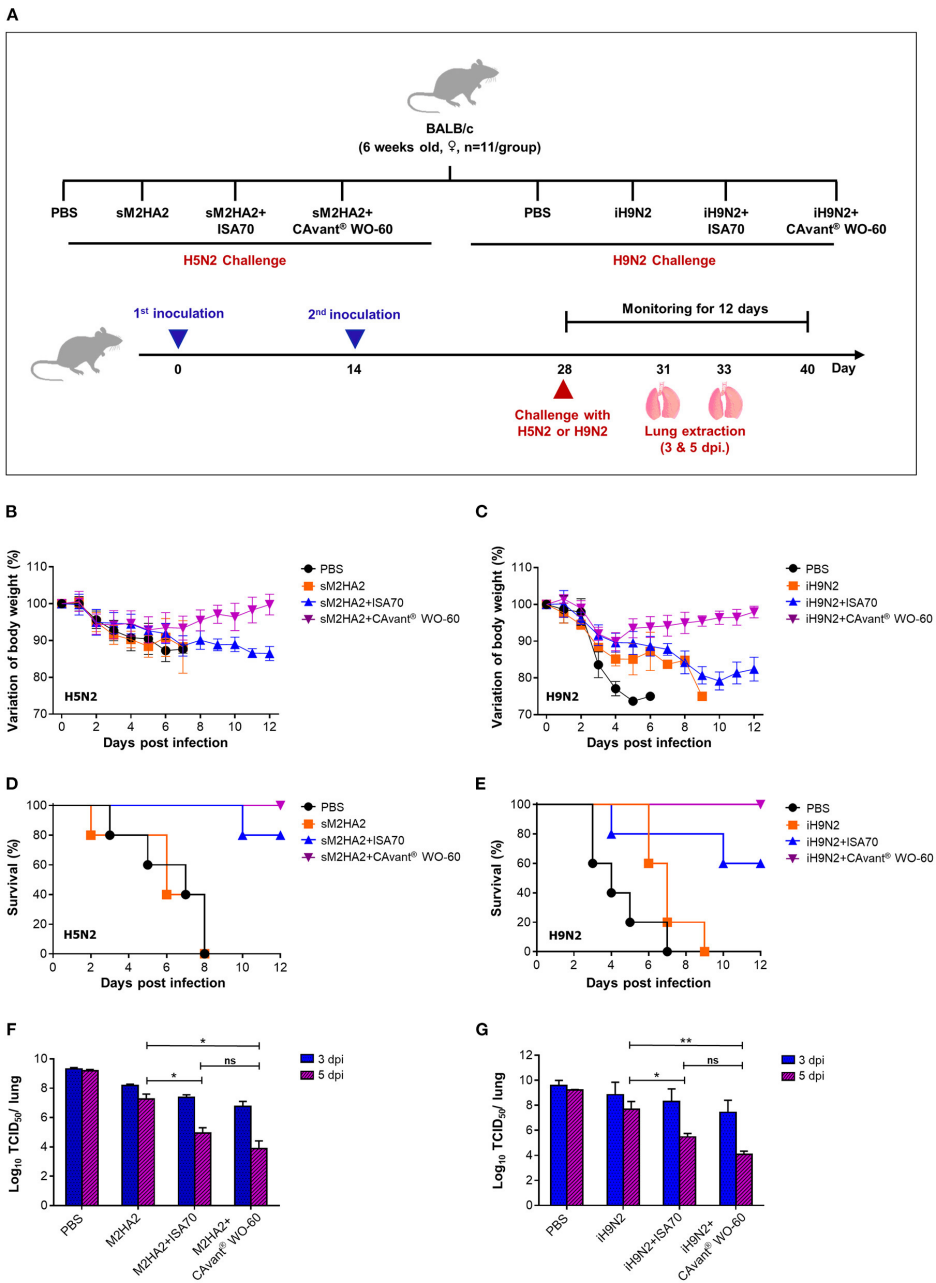
Protective efficacy against lethal influenza virus infection in mice. **(A)** Schematic depiction of mice experiment strategy. BALB/c mice were intramuscularly administrated twice every other week. Mice were intranasally challenged with 10LD50 of mouse-adapted A/Aquatic bird/Korea/W81/2005(H5N2) and A/Chicken/Korea/01310/2001(H9N2) at 2 weeks after the second immunization. **(B,C)** The changes in body weight and **(D,E)** survival rate after performing lethal challenges with H5N2 and H9N2, respectively, were monitored for 12 days. **(F,G)** Virus titers in the lung tissues were investigated at 3 and 5 DPI by TCID50 in the MDCK cell following infection with A/Aquatic bird/Korea/W81/2005(H5N2) and A/Chicken/Korea/01310/2001(H9N2), respectively. The bar shows the mean ± SD of *n* = 5 or *n* = 3 samples. Data from one representative experiment of two independent experiments are shown. Comparison of groups was analyzed by ANOVA and Tukey's multiple comparisons test. *p < 0.05, **p < 0.01 between CAvant® WO-60 adjuvanted, ISA 70 VG adjuvanted, and antigen-only group.

The finding that CAvant® WO-60 effectively induced both humoral and cellular immune responses to AIV antigens suggested that this adjuvant might improve the ability of the sM2HA2 or iH9N vaccine to protect mice against lethal AIV infection. Mice were therefore immunized i.m. twice at a 14 day interval with sM2HA2 or iH9N2, either alone or emulsified in CAvant® WO-60 or ISA 70 VG adjuvant. Mice immunized with sM2HA2 were challenged on day 28 with 10LD50 of A/Aquatic bird/Korea/W81/2005(H5N2) influenza virus, whereas mice immunized with iH9N2 were challenged on day 28 with 10LD50 of A/Chicken/Korea/116/2004 (H9N2) influenza virus. The efficacy of the immunization group was assessed by measuring weight loss and survival for up to 12 days post-infection (DPI) (Figure 3A).

Mice injected with PBS or with sM2HA2 alone without an adjuvant showed significant loss in body weight (over 25%), with 100% dying by 8 days. By contrast, all the mice that were immunized with sM2HA2 emulsified in CAvant® WO-60 were protected against challenge, with little weight loss until day 7, followed by gradual recovery. The mice that were immunized with sM2HA2 emulsified in ISA 70 VG exhibited continuous body weight loss, with 80% surviving until day 12 (Figures 3B,D). Similarly, mice injected with PBS or with iH9N2 alone without an adjuvant showed significant loss in body weight after the challenge, with all dying on days 7–9. By contrast, mice that were immunized with iH9N2 emulsified in CAvant® WO-60 exhibited little weight loss, with all surviving for 12 days after the challenge. Body weight loss was significantly greater in mice immunized with iH9N2 emulsified in ISA 70 VG than in mice immunized with iH9N2 emulsified in CAvant® WO-60, with 40% of the former dying following challenge with the virus (Figures 3C,E). Taken together, these results suggest that immunization using the CAvant® WO-60 adjuvant can improve the protective efficacy of vaccines against influenza virus infection.

### CAvant® WO-60 Adjuvant Augmented the Ability of AIV Vaccines to Control Lung Virus Titers After Challenge

To better understand the superior protection provided by the AIV vaccines when they were emulsified in CAvant® WO-60 adjuvant, randomly selected challenged mice from the above groups were sacrificed 3 or 5 days after infection, and the virus titers in their lungs were measured using the TCID_50_ method. Compared with mice injected with PBS or with sM2HA2 alone without adjuvant, the mice that were vaccinated with sM2HA2 emulsified in CAvant® WO-60 had significantly lower virus titers in the lungs 5 days after challenge. These titers were also lower than in the lungs of mice immunized with sM2HA2 emulsified in ISA 70 VG (Figure 3F).

Similar results were obtained when the iH9N2-immunized mice were examined, with virus titers being significantly lower in the lungs of mice immunized with iH9N2 emulsified in CAvant® WO-60 adjuvant than in the lungs of mice injected with PBS or with iH9N2 alone without an adjuvant. Virus titers were also lower in the lungs of mice immunized with iH9N2 emulsified in CAvant® WO-60 than in the lungs of mice immunized with iH9N2 emulsified in ISA 70 VG (Figure 3G). These results suggest that CAvant® WO-60 can significantly improve the ability of AIV vaccines to inhibit viral replication in the lungs, thereby providing superior protection from infection with the influenza virus.

### The CAvant® WO-60 Adjuvant Improves Antigen-Specific Seroconversion Responses of The NDV-H9N2 Bivalent Vaccine

**Figure 4:**
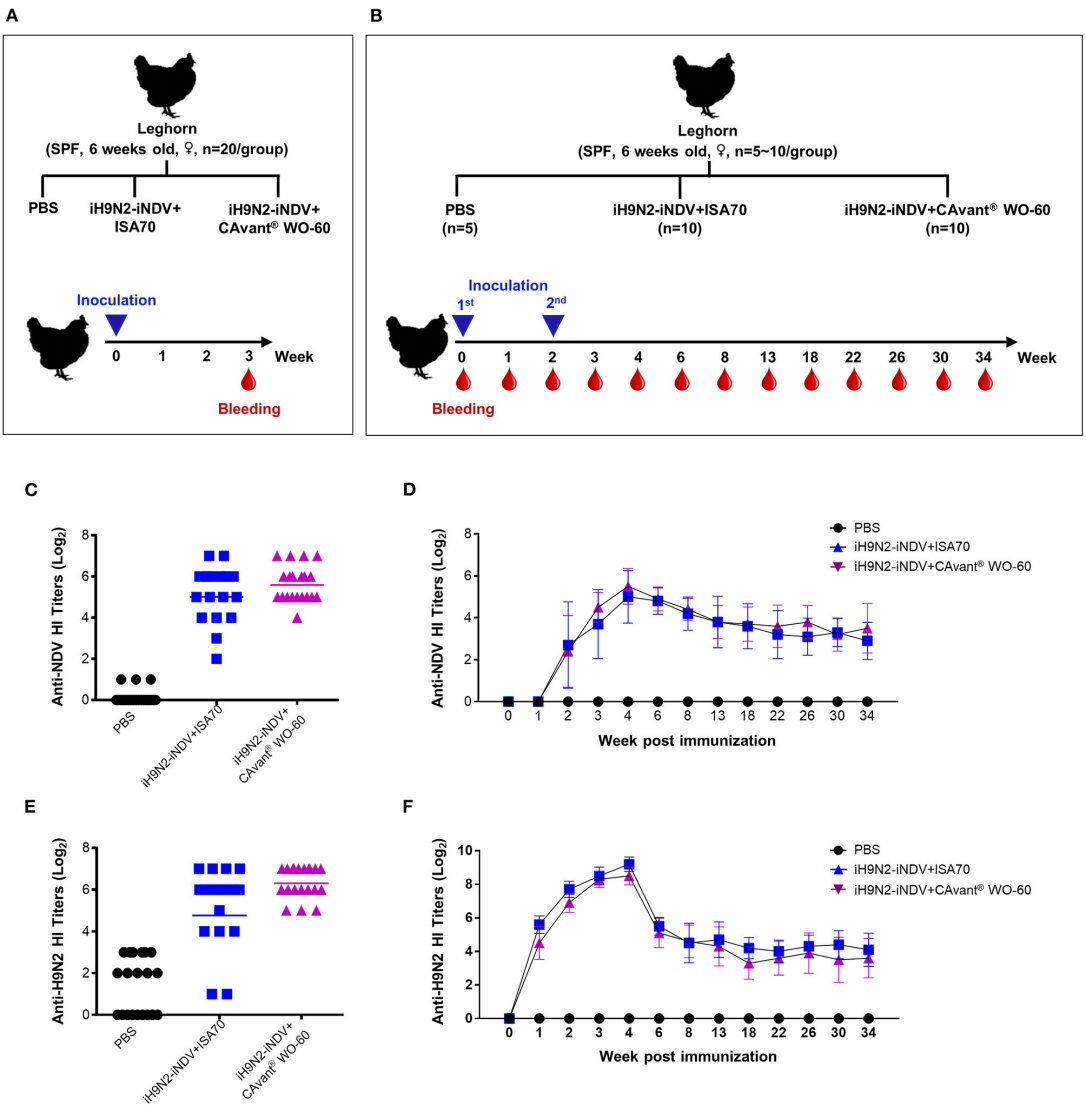
Evaluation of the antigen-specific hemagglutination inhibition (HI) titers following immunization of chicken. **(A,B)** Schematic depiction of chicken experiment timeline. SPF chickens were intramuscularly immunized with one or two doses of bivalent iH9N2 and iNDV antigen either with ISA 70 VG or CAvant® WO-60 adjuvant or PBS at weeks 0 and 2. Geometric means of hemagglutination inhibition antibody titers to NDV LaSota in immunized chickens with **(C)** one dose and **(D)** two doses of vaccine. Geometric means of hemagglutination inhibition antibody titers to H9N2 in immunized chickens with **(E)** one dose and **(F)** two doses of vaccine. Data presented as geometric mean (GM) of *n* = 20 and bar shows the mean ± SD of *n* = 10 or *n* = 5 samples.

To further assess the effect of the CAvant® WO-60 adjuvant on host animals, chickens were immunized i.m. once (priming) or twice (boosting) with a bivalent vaccine consisting of iNDV plus iH9N2 emulsified in CAvant® WO-60 or ISA 70 VG at a ratio of 3:7 (v/v). Control chickens were injected with PBS, and serum samples were collected from individual birds to determine their homologous HI titers (Figures 4A,B).

Twenty-one days after a single injection, the serum geometric mean titers (GMT) of anti-HI antibodies specific for NDV LaSota (1:50.2) and H9N2 (1:81.5) viruses from birds immunized with iNDV plus iH9N2 emulsified in CAvant® WO-60 surpassed the minimum protective titers (≥1:40). By contrast, titers in birds immunized with these viruses emulsified in ISA 70 VG were close to, but did not reach, the minimum protective titers. None of the control chickens was seropositive (≥1:4) against either virus (Figures 4C,E).

The kinetics of antigen-specific antibody induction and persistence were determined in immune sera collected at different times from chickens that received both priming and boosting immunizations. Fourteen days after the priming immunization, the mean HI titer for NDV LaSota virus was seroconverted in chickens from both adjuvant groups. The mean HI titers were further increased after the booster immunization, reached their maximum (>1:32) 14 days after the booster immunization, and declined gradually thereafter. Mean HI titers for NDV LaSota virus throughout the examination period were comparable in chickens immunized with antigens emulsified in CAvant® WO-60 and ISA 70 VG adjuvants (Figure 4D).

Seven days after the first immunization, the mean GMT HI titer for the H9N2 virus was seroconverted in both adjuvant groups. These titers were further increased after the booster immunization, maximizing at >1:128 14 days after the booster immunization. Similar to the NDV LaSota HI titer kinetics, H9N2 HI titer also decreased over time, with mean HI titers for H9N2 virus comparable throughout the examination period in chickens immunized with antigens emulsified in CAvant® WO-60 and ISA 70 VG adjuvants (Figure 4F). Taken together, these results suggest that, compared with the reference adjuvant ISA 70 VG, CAvant® WO-60 provides comparable or even superior immune responses to avian antigens and protection against avian viruses.

## Discussion

Poultry accounts for a significant share of worldwide food production (24). Effective protection of the global poultry industry against costly infectious diseases requires proper monitoring and active health management of the birds. Currently, active immunization using various types of infectious agents is a routine practice (22). Adjuvants contribute to the effectiveness of vaccines by enhancing the immunogenicity of antigens (17, 18). Because hundreds of poultry vaccines are produced annually, even small improvements in vaccine efficacy will have enormous dividends. Adjuvants can enhance vaccine efficacy in several ways, including by reducing the number of booster injections, reducing the amount of antigen per injection, broadening immune responses to antigenic variants, increasing vaccine availability, and reducing vaccine price.

Oil-based adjuvants have been shown to be superior to alternative formulations in vaccinating poultry. In particular, the W/O adjuvants ISA 70VG and ISA 71VG, which perform similarly, were reported to be for poultry vaccines (25). ISA 70VG-based vaccines against inactivated AIV and NDV induced higher protective antibody titers than Al (OH) 3/mineral oil-based vaccines (26, 27). ISA 71VG is effective at eliciting humoral and cellular immune responses with the potential to generate protective immunity against Eimeria and NDV (28–30).

CAvant® WO-60 is a novel W/O emulsion type adjuvant containing a high-grade injectable mineral oil, refined non-ionic hydrophilic and non-ionic lipophilic surfactant system. The small average diameter and low viscosity of CAvant® WO-60 facilitate syringeability and injectability. *In vivo* safety profiles in chicken demonstrated no abnormal clinical sign or bodyweight reduction after vaccination (Supplementary Table 1 and Supplementary Figure 1). The CAvant® WO-60 emulsion can have a variety of effects on vaccine biological activity by modulating antigen delivery to APCs or modulating a slow release of antigen to continue the stimulation of the immune system or having an intrinsic adjuvant effect through direct stimulation of immune cells. In addition, CAvant® WO-60 can have immunomodulatory properties by directing balance immune responses toward T helper (TH) 1 and TH2 response (18, 31).

Simultaneous enhancement of antibody and cell-mediated immune responses remains the prime objective of vaccination. Apart from virus neutralization, antibody-induced host effector mechanisms that aid in the clearance of virus are important properties of influenza vaccines (32–34). Vaccines consisting of viral antigens emulsified in CAvant® WO-60 induced better but not significantly higher level of antigen-specific IgG1 and IgG2a responses than vaccines consisting of the same antigens emulsified in ISA 70VG. Generally, Th1 cells improve the production of IgG2a, which effectively neutralizes and clears viruses, whereas Th2 cells induce IgG1 Abs, which effectively neutralize viruses (34). These results indicate that the CAvant® WO-60 adjuvant mediates the Th1/Th2 cell balance, providing effective cellular and humoral immune responses to antigens contained in these vaccines.

This study also investigated the splenic recall of Th1 and Th2 cell signature cytokine responses to the vaccine antigens by ELISPOT. Under these experimental conditions, antigens emulsified in CAvant® WO-60 increased but not at a significantly higher level of the frequencies of antigen-specific IFN-γ- and IL-4-secreting T cells compared to the antigens emulsified in ISA 70VG; the results showed good agreement with the IgG1 and IgG2a antibody titers. Furthermore, injection of antigens emulsified in CAvant® WO-60 completely protected mice from lethal challenges with A/Aquatic bird/Korea/W81/2005 (H5N2) and A/Chicken/Korea/116/2004 (H9N2) viruses, suggesting that CAvant® WO-60 can be an effective adjuvant for vaccines that target different influenza A virus subtypes. To further assess the potency of this adjuvant, the effects of CAvant® WO-60 on lung virus titer was assessed following lethal challenge with A/Aquatic bird/Korea/W81/2005 (H5N2) and A/Chicken/Korea/116/2004 (H9N2) viruses. Immunization with antigens emulsified in CAvant® WO-60 reduced the virus titers in the lungs after lethal challenges with these influenza A subtypes.

Although CAvant® WO-60 showed better adjuvant efficacy than ISA 70 VG in mice immunization experiments, the two adjuvants showed similar efficacy in chickens. Chickens were immunized with bivalent inactivated NDV and AIV emulsified in CAvant® WO-60 or ISA 70 VG, and their HI antibody titers, which correlate positively with protection in chickens, were evaluated (35). Chickens subjected to a single immunization with inactivated viruses in CAvant® WO-60 were found to have seroprotective levels of GM HI antibody titers 3 weeks after immunization, 1:50.2 against NDV and 1:81.5 against H9N2. Seroprotective GMT against these antigens was not achieved in chickens immunized with inactivated viruses emulsified in ISA 70 VG. Chickens subjected to both primary and booster immunizations were monitored for HI antibody responses for up to 32 weeks after the initial immunization. HI antibody kinetics were found to be comparable in chickens that received two injections of antigens emulsified in CAvant® WO-60 or ISA 70 VG adjuvant, with titers against both NDV and H9N2 peaking 4 weeks after the initial immunization and these antibody titers remaining seropositive for at least 32 weeks. Taken together, the results suggested that the two adjuvants were similarly effective in generating HI antibody responses.

In conclusion, this study demonstrated that the novel water-in-oil emulsion CAvant® WO-60 adjuvant was capable of inducing strong mixed humoral and cellular immune responses against viral antigens. These responses were capable of protecting mice against lethal influenza challenge, as well as enhancing the seroprotective antibody responses against the NDV and AIV bivalent antigen in chickens. The adjuvant efficacy of CAvant® WO-60 was comparable to that of the poultry vaccine adjuvant ISA 70VG. These findings suggest that CAvant® WO-60 adjuvant would be a promising candidate for the development of an effective poultry vaccine.

## Data Availability Statement

The original contributions presented in the study are included in the article/Supplementary Material, further inquiries can be directed to the corresponding authors.

## Ethics Statement

The animal study was reviewed and approved by Institutional Animal Care and Use Committee of Chungnam National University, Daejeon, Republic of Korea (Reference number CNU-00952) and ChoongAng Vaccine Laboratories Co., Ltd., Daejeon, Republic of Korea (Reference number CAVAC 18-009).

## Author Contributions

J-SL and S-SY: conceived and designed the experiments. E-SL, Y-JS, WC, and Y-HA: performed the experiment. E-SL, Y-JS, I-JY, J-SL, and S-SY: analyzed the data and wrote the paper. All authors contributed to the article and approved the submitted version.

## Funding

This work was supported by the Ministry for Food, Agriculture, Forestry and Fisheries (Grant No. 316043-3), Republic of Korea.

## Conflict of Interest

Y-JS, Y-HA, I-JY, and S-SY were employed by company Choong Ang Vaccine Laboratories Co., Ltd. The remaining authors declare that the research was conducted in the absence of any commercial or financial relationships that could be construed as a potential conflict of interest.

## Publisher's Note

All claims expressed in this article are solely those of the authors and do not necessarily represent those of their affiliated organizations, or those of the publisher, the editors and the reviewers. Any product that may be evaluated in this article, or claim that may be made by its manufacturer, is not guaranteed or endorsed by the publisher.
