# Supplementary material for: CAvant® WO-60 as an Effective Immunological Adjuvant for Avian Influenza and Newcastle Disease Vaccine
Source: Front Vet Sci. 2021 Dec 3;8:730700. doi: 10.3389/fvets.2021.730700 (PMC8677964; doi:10.3389/fvets.2021.730700)
Supplement: Supplementary file 1 [file Data_Sheet_1.docx]

**Supplementary Materials**: In vivo safety profile of the CAvant^®^ WO-60

**TABLE S1:** Clinical signs after vaccination

| **Group** | **Clinical signs**  **(No of birds positive/No of birds examined)** |
| --- | --- |
| Test group | 0/5 |
| Control group | 0/5 |


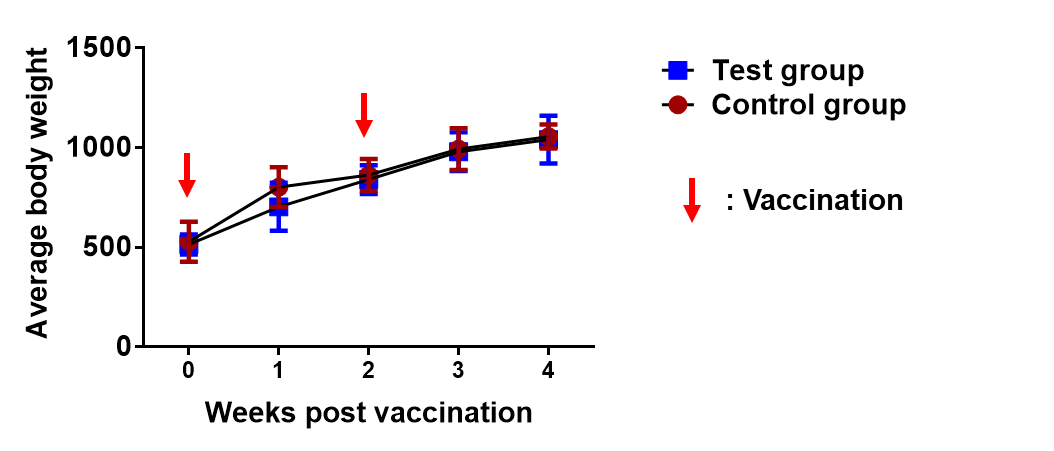


**FIGURE S1.** Bodyweight variation after vaccination. Chickens in the test group were vaccinated intramuscularly twice every other week with 500 µl of inoculum containing 10^8^ EID_50_/dose from Newcastle disease virus (LaSota) and 10^8^ EID_50_/dose from LPAIV (A/chicken/Korea/01310/2001) antigen emulsified with CAvant® WO-60. Chickens in the control group were maintain without vaccination. The bodyweight of the chickens was measured at 0, 1, 2, 3, and 4 weeks after vaccination. Error bars indicate the mean ± SD of n=5 samples.
